# Standardizing admission and discharge processes to improve patient flow: A cross sectional study

**DOI:** 10.1186/1472-6963-12-180

**Published:** 2012-06-28

**Authors:** Berta Ortiga, Albert Salazar, Albert Jovell, Joan Escarrabill, Guillem Marca, Xavier Corbella

**Affiliations:** 1 Clinical Services, Hospital Universitari de Bellvitge IDIBELL, L’Hospitalet de Llobregat, Barcelona, 08907, Spain; 2 Hospital Universitari de Bellvitge IDIBELL, C. Feixa Llarga s.n, L’Hospitalet de Llobregat, Barcelona, 08907, Spain; 3 Universidad Autónoma de Barcelona, Fundació Josep Laporte, Barcelona, 08041, Spain; 4 Health Department, Institut d’Estudis de la Salut, Barcelona, 08005, Spain; 5 Universidad de Vic, Vic, 08500, Spain; 6 Hospital Universitari de Bellvitge, IDIBELL, L’Hospitalet de Llobregat, Barcelona, 08907, Spain

**Keywords:** Patient flow, hospitals, practice, management

## Abstract

**Background:**

The aim of this study was to evaluate how hospital capacity was managed focusing on standardizing the admission and discharge processes.

**Methods:**

This study was set in a 900-bed university affiliated hospital of the National Health Service, near Barcelona (Spain). This is a cross-sectional study of a set of interventions which were gradually implemented between April and December 2008. Mainly, they were focused on standardizing the admission and discharge processes to improve patient flow. Primary administrative data was obtained from the 2007 and 2009 Hospital Database. Main outcome measures were median length of stay, percentage of planned discharges, number of surgery cancellations and median number of delayed emergency admissions at 8:00 am. For statistical bivariate analysis, we used a Chi-squared for linear trend for qualitative variables and a Wilcoxon signed ranks test and a Mann–Whitney test for non-normal continuous variables.

**Results:**

The median patients’ global length of stay was 8.56 days in 2007 and 7.93 days in 2009 (p < 0.051). The percentage of patients admitted the same day as surgery increased from 64.87% in 2007 to 86.01% in 2009 (p < 0.05). The number of cancelled interventions due to lack of beds was 216 patients in 2007 and 42 patients in 2009. The median number of planned discharges went from 43.05% in 2007 to 86.01% in 2009 (p < 0.01). The median number of emergency patients waiting for an in-hospital bed at 8:00 am was 5 patients in 2007 and 3 patients in 2009 (p < 0.01).

**Conclusions:**

In conclusion, standardization of admission and discharge processes are largely in our control. There is a significant opportunity to create important benefits for increasing bed capacity and hospital throughput.

## Background

At the moment, hospitals face an increasing demand for hospitalization, for medical staff due to the introduction of innovative technology in diagnostic and therapeutic procedures, for higher standards in clinical safety and, finally, an increasing patient demand for better quality services [1,2]. Optimal bed management is a strategic aim in any hospital as the provision of an inpatient bed, together with the staff and supplies involved, accounts for much of its most complex and expensive activity. The way beds are managed affects the way other hospital departments perform since many are dependent on bed availability, such as emergency services, operating theatres, etc. At the same time, these other hospital departments have an impact on bed usage [3]. Therefore, it is essential to have an efficient and correct bed management in order to improve service delivery.

From patient experience, an admission to a bed as an inpatient in an acute hospital is a major event, independent of this admission being an emergency or from a waiting list. First of all, patient experience will depend on the availability of beds. That is to say, that when patients need an emergency admission, it is important to be admitted quickly and to an appropriate bed, avoiding unnecessary waiting times in the emergency room. On the other hand, if patients are being admitted from a waiting list for elective surgery, it is important to minimize the number of occasions that admissions are cancelled as a result of there being no bed available [4].

The hospitalization process has three main stages: an admission, an inpatient period and a final stage with the discharge process. An inefficient bed management in any of the three stages of the hospitalization can cause a mismatch between demand and capacity. It has been proved that when bed demand exceeds capacity, patient admissions and scheduled surgical procedures can be delayed or cancelled. Traditionally, it has been assumed that the variability in the demand comes from the emergency patient. Interventions focused primarily on emergency departments have had limited success [5]. However, repeated case studies have shown that elective admissions are often the major cause of variation as they are more unpredictable than the emergency admissions [6,7]. In addition, the greatest variation is typically in the number of discharges and, therefore, efforts to reduce variation should start with the discharge process and not in the admission process [8]. Then, to have information about planned discharges 24-h in advance would allow a higher planning and an optimal bed assignment. Moreover, the discharge process should start at the point of admission in the case of planned admissions, as in some cases the estimated length of stay without a medical complication is known. Discharge planning allows for a better and quicker bed assignment in hospitals and the development of nurses and other staff working in discharge coordinator roles [9]. In this sense, it has been proved that multidisciplinary teams can improve the delivery of health services and patient care [10-12]. All admissions and discharges of the hospital should be centrally managed [13] and planned, as single-department solutions may create or worsen bottlenecks in other areas.

During the hospitalization process, patient flow is a strategic aim for the healthcare enterprise. Hospitals can combine process management with information technology to redesign patient flow for maximum efficiency and clinical outcomes. Information is the foundation of any patient flow initiative. Patient flow is built upon the capture, integration and sharing of information, both within and across the different departments and staff [14]. This critical foundation can be immensely challenging to hospitals both with numerous information systems and departments that operate as silos [15]. Actionable information triggers patient care events and enables automated reminders. The aim of this study was to evaluate how hospital capacity was improved through focusing on standardizing the admission and discharge processes.

## Methods

This study was set in a 900-bed university affiliated hospital located in the metropolitan area of Barcelona (Spain) that belongs to the National Health System. It attends more than 120,000 emergency visits annually and the mean number of monthly elective admissions is 1,650 (95% CI 1,609 to 1,691), not taking into account day surgery. For our study, we created an interdisciplinary team of clinicians, hospital administrators and patients/families to examine bottlenecks and improvement areas in service delivery. We then selected high impact interventions focused on reducing the variation in the admission process for elective admissions, avoiding unnecessary cancellations of surgery interventions that have an impact on waiting lists, and on planning and standardizing the discharge process. All the interventions were implemented between April and December 2008. See Table 1 for intervention lists.

**Table 1:** Intervention List

**Discharge process management interventions:**	**Admission process management interventions:**
▪ Enhance multidisciplinary teamwork: doctor, nurse, house officer and central admissions unit.	▪ Bed Management by a central admissions team planning and scheduling patient flows: right patient, right place and right time.
▪ Set a planned date for discharge on day of admission or at pre-admission, using protocols for common conditions with <72-h expected length of stay.	▪ Central admissions in a Surgery Admission Unit.
▪ Discharge planned 24-h in advance for >72-h expected length of stay.	▪ Patients admitted on the same day of surgery.
▪ Nurse-led discharge.	▪ Enhance day-surgery rates of selected processes.
▪ Plan discharge needs: discharge report, pharmacy prescriptions, sanitary transport, home care, etc.	▪ Avoid “on the day” cancellations of elective patients.

Standardization of the admission process included admission on the same day as surgery and promoting day-surgery rather than inpatient care, both aimed to free up bed days for emergency admissions and to admit major elective patients from a waiting list. To promote planning discharges 24-h in advance consisted in educating the clinicians on entering the discharge information in the electronic patient report. Then the house officers daily worked together with the physician in order to plan the discharge of the patient: discharge report, pharmacy prescriptions, the need of transportation to home, etc. At the same time, the nurse became the patient manager as he or she knew the discharges for the following day and that allowed an optimized task organization for the day, to identify possible home care arrangements for the patient, to collect patient documents from the house officer, and personally give them to the patient so that the patient could ask about any possible doubts. At the same time, the patient/family did not need to go personally to the house officer to collect the information and could get more feedback from their nurse manager. When the patient left the bed, the nurse entered the information in the system, which also prevented the patient/family to personally go and communicate their discharge to the admission unit when leaving the hospital. Bed management was done through a centralized team, with the help of the Information System, which placed emergency and elective patients in the most appropriate beds, allowed patient transfers between wards and checked patient discharge status, in order to have a correct patient allocation and a global vision of the hospital occupancy at all times.

For this study, we included all patients admitted to hospital wards before the multi-intervention, between the 1^st^ of January and the 31^st^ of December 2007, and after the implementation, between the 1^st^ of January and the 31^st^ of December 2009.

The following variables were recorded through the Hospital General Database: patient demographics, main diagnosis and procedure, admission and discharge dates, date of surgery, number of emergency patients waiting for a bed at 8:00 am, causes of patient cancellation, percentage of planned discharges 24-h in advance, number of patient outliers and number of day-surgery interventions. We did not look for ethical approval, as the organizational change described in this study did not cause any change in the clinical management of the patients and did not make any intervention to the individual patient.

The main outcome measures were: median length of stay, proportion of patients admitted on the same day of surgery, percentage of planned discharges, number of surgery cancellations, proportion of day-surgery, median number of delayed emergency admissions at 8:00 am due to lack of bed and median number of patient outliers, risk-adjusted mortality rate and risk-adjusted readmissions rate.

To describe categorical variables we used the total number of cases (N, days) and the percentage of each category and we used the Chi-squared for linear trend in bivariate analysis. All continuous variables were expressed as median ± interquartile range, and changes were assessed using the Wilcoxon signed ranks test and the Mann–Whitney test. A *P* value of less than 0.05 was considered statistically significant. All statistical analysis was conducted using the Statistical Software Program [16] for Windows (version 14).

## Results

We included 53,361 admissions, of which 27,784 were done in 2007 and 28,577 were done during 2009. Table 2 shows the general activity information during these two years, 2007 and 2009. The number of patient admissions for scheduled surgery was 13,824 patients in 2007 and 14,548 patients in 2009. The proportion of patients admitted on the same day of surgery significantly increased, from 64.87% in 2007 to 86.01% in 2009 (p < 0.05) (Table 3). The patients’ global length of stay was 8.56 days in 2007 and 7.93 days in 2009, without day surgery patients. The scheduled admitted patients length of stay was 4.85 days in 2007 and 4.54 days in 2009, especially caused by the “same day admission” policy implemented, as the pre-surgery length of stay was reduced from 0.58 days in 2007 to 0.26 days in 2009 (p < 0.05). The number of cancelled interventions due to lack of beds was 216 patients in 2007 and 42 patients in 2009. The median number of day-surgery interventions per day increased, especially due to the increase in day-case rates for the procedures: knee arthroscopy, varicose veins and bunions (Table 2).

**Table 2:** General hospital data during years 2007 and 2009

	** 2007**	** 2009**	***P *value**
	**Median (IQR: Q1-Q3)**	**Median (IQR: Q1-Q3)**	
Available hospital beds	776.00 (724.00-819.00)	757.00 (699.50-790.00)	<0,01
Emergency daily visits	344.00 (319.00-367.00)	337.00 (307.00-361.00)	<0.01
All scheduled admissions (including day surgery)*	59.00 (20.00-85.00)	64.00 (10.00-91.00)	0.78
Scheduled hospital admissions*	45.50 (13.00-69.00)	47.00 (8.50-75.00)	0.63
Emergency admissions	36.00 (31.50-41.00)	36.00 (31.00-40.00)	0.24
Day surgery admissions*	13.00 (0–23.00)	16.00 (0–24.50)	<0.05
Hospital occupancy	87.37 (87.29-88.64)	91.8 (89.70-94.05)	<0.01

*Considering 365.25 days per year.

**Table 3:** Main Key Performance Indicators during years 2007 and 2009

	**2007**	**IQR: Q1-Q3**	**2009**	**IQR: Q1-Q3**	***P *value**
Same day of surgery admission	64.87%	51.07% to 70.02%	86.01%	83.50% to 88.93%	<0.05
Pre-surgery length of stay (days)*	0.58	0.53 to 0.70	0.26	0.24 to 0.32	<0.05
Global length of stay (without day surgery, days)*	8.56	6.88 to 10.01	7.93	6.78 to 9.51	0.051
Scheduled patient length of stay (without day surgery, days)*	4.85	3.73 to 6.33	4.54	3.62 to 4.54	<0.05
A&E patient length of stay (days)*	11.64	9.82 to 13.93	11.46	9.49-13.56	0.22
Cancelled interventions	216	_	42	_	_
A&E patients admitted to hospital	10.46%	9.26% to 11.90%	10.49%	9.20% to 12.13%	0.33
Discharge planning	43.05%	40.09% to 45%	86.01%	84.92% to 87.10%	<0.01
Daily patients placed out of service	70	56 to 78	62	49 to 69	<0.05
Emergency inpatients waiting for a bed	5	1 to 11	3	1 to 7.50	<0.01

*Mann–Whitney test.

**Figure 1:**
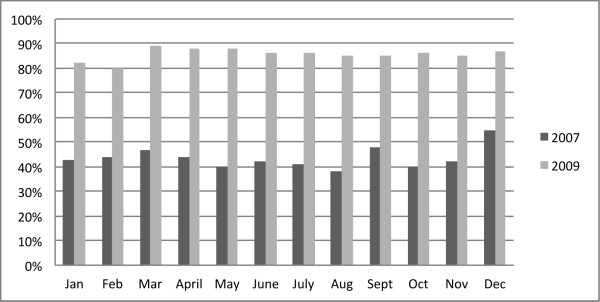
Comparison of percentage of planned discharges during 2007 and 2009, by months.

The standardization of the discharge process was based on discharge planning and teamwork building (Figure 1). In this sense, the median number of planned discharges went from 43.05% in 2007 to 86.01% in 2009.

The median number of patients placed out of service in 2007 was 70 patients and 62 patients in 2009 (p < 0.05). That is to say, the percentage of inpatient outliers diminished from 9.71% in 2007 to 7.30% in 2009 (p < 0.05). The median number of emergency patients waiting for an in-hospital bed at 8:00 am was 5 patients per day in 2007 and 3 patients per day in 2009 (P < 0.01). The percentage of emergency visits that were finally admitted to the hospital was 10.46% in 2007 and 10.49% in 2009. The percentage of emergency admissions over global admissions was 50.19% in 2007 and 49.10% in 2009. Risk-adjusted mortality rate diminished from 1.02 in 2007 to 0.89 in 2009 [17] (Table 4).

**Table 4:** Quality indicators during years 2007 and 2009

	**2007**	**2009**
Readmissions Rate	7.3%	7.4%
Risk-adjusted Readmissions Rate*	1.07	1.04
Complications Rate	5.2%	6.8%
Risk-adjusted Complications Rate*	0.96	1.16
Mortality Rate	5.1%	4.6%
Risk-adjusted Mortality Rate *	1.02	0.89

* Comparison group created from Peer hospitals, data from years 2009 and 2010 [17].

## Discussion

The optimization of hospital care resources by managing variation in the admission and discharge processes has proven to be effective. This multiple intervention project increased hospital productivity. Firstly, the main consequence due to the admission process has been the reduction of the length of stay, especially in scheduled admissions due to the reduction in the pre-surgery stay as a high percentage of patients were admitted on the same day as surgery. In addition, day surgery was considered as the first option for some surgery processes. Secondly, the significant increase in planned discharges helped sharing information among staff and enhanced teamwork. House officers were able to prepare all the information and patient arrangements for the day. In addition, patients and their families awaited comfortably in their rooms instead of being the messengers of information among the hospital silos. However, the implementation of these high impact changes required leadership, multidisciplinary teamwork and board level commitment as they affected the whole organization. All interventions were based on “lean” concepts, basically to reduce waste in terms of human resources, public health services and patient quality of care as well as to gain flexibility in hospital capacity.

Interventions included in this study are mostly dependent on the leadership and control of the management team [18] in order to assess the appropriateness of acute bed usage. There is an opportunity by process reengineering to increase bed capacity and productivity with the same fixed costs. In this sense, actions that lead to an increase of productivity without diminishing the service quality, or even increasing it, should be considered as successful key factors for best practices and a competitive advantage for any hospital. Bed management issues therefore warrant high consideration within the hospital’s management team. Some Boards have recognised the importance of hospital operations and that the person in charge of this area of management should be a senior member of the hospital’s executive committee.

In our study we have seen how redesigning operational aspects of the care delivery process that do not affect quality of care, can reduce scheduled admissions cancellations and the number of emergency admissions waiting for a bed. It is crucial not to block beds for elective admissions in advance, as supply of available beds will come through the discharges of the day. The way beds are managed has consequences on all organization levels: emergency and accident departments, surgery theatres, as in both cases their activity depends upon bed availability. However, there are many other aspects to consider when analysing bed capacity such as its efficient use. Departments that are inefficient can lengthen hospital stays and use beds unnecessarily [19].

Around 50 per cent of hospital admissions involve non-emergency patients who have been on a waiting list, mostly for a surgical operation. Waiting dominates many citizens’ perceptions of hospital care. While they are waiting, patients may be in considerable pain and discomfort and this interferes with their normal lifestyle and it adds to the workload of primary care [20]. On the other hand, in order to avoid last moment surgery cancellations due to lack of beds, a lot of professionals are likely to admit their patients the day before surgery and waste a one-day bed unnecessarily. It is then important to reach a consensus between the physicians and the management team in order to maximize profit for both parties, including patients and their families. The intervention for scheduled surgery consisted in a surgery admission unit [21] where the patient was admitted on the same day as surgery and was prepared without being given a bed. In this context, when patients were admitted each morning there were not any free beds in hospital wards, and they had to wait until other patients left the hospital. A possible drawback was that there could be a delay in bed assignments, which could have an impact on the rotation of patients in recovery theatres after the surgery and then in operating theatre flows.

In our hospital we reached 85% of planned discharges (Figure 1). Delayed discharge triggered waits on trolleys in the emergency room and in operating theatres. Planning ensured an early and certain discharge as well as a better bed assignment because there was information about which beds would be available. Therefore, the number of patient outliers in the hospital significantly diminished. A limitation of planning discharge was that not all of them were effectively real the following day. The percentage of cancelled discharges was usually less than 10%. However, the importance of the planning was precisely to avoid improvisation of all the staff that participated in the discharge: physician, nurse, house officer, sanitary transport, families and patients and others. In fact, discharge process should start in the admission point, as it is the mismatch between demand and supply of beds that promotes delays and bottlenecks in the system [8,22].

Another limitation of our study was that this multi-intervention was only implemented in one hospital, so the study’s generalizability is limited. In our experience, it is crucial that management leaders focus on efforts to promote admission on the same day as surgery and to promote an early hospital discharge so that other patients can be placed in the most appropriate bed as soon as possible.

## Conclusion

In conclusion, admission and discharge standardization and therefore length of stay are largely in our control. There is a significant opportunity to redesign patients’ pathways and improve patient flow to create important benefits for bed management and hospital throughput, which ultimately improve quality and the safeness of patient care.

## Competing interests

There are not any financial and non-financial competing interests in relation to this manuscript.

## Authors’ contributions

BO contributed to conception and design, acquisition of data, performed the statistical analysis and interpretation of data, as well as drafting the manuscript and adding all the comments from other authors; AS contributed to conception of the study as well as to the interpretation of data and to drafting the discussion of the manuscript; AJ and JE contributed to revising the manuscript critically for important intellectual content; GM participated in the conception, program design and in revising the draft manuscript; XC participated in revising the manuscript critically for important intellectual content. All authors read and approved the final manuscript.

## Pre-publication history

The pre-publication history for this paper can be accessed here:

http://www.biomedcentral.com/1472-6963/12/180/prepub

## References

[B1] Hendrich AL Lee N Intra-Unit Patient Transports: Time, Motion, and Cost Impact on Hospital Efficiency Nurs Econ 2005 23 157 64 16189980

[B2] Allder S Silvester K Walley P Understanding the current state of patient flow in a hospital Clin Med 2010 10 441 4 21117373 10.7861/clinmedicine.10-5-441 PMC4952402

[B3] Management B Review of national findings 2003 Audit Commission, National Health Service of England and Wales

[B4] Management of admission in acute hospitals Review of the national findings 2006 Healthcare Commission, National Health Service of England and Wales

[B5] Howell E Bessman E Kravet S Kolodner K Marshall R Wright S Active bed management by hospitalists and emergency department throughput Ann Intern Med 2008 149 804 11 19047027 10.7326/0003-4819-149-11-200812020-00006

[B6] Tamames S Perez Rubio A Castrodeza Sanz J Canton Alvarez MB Luquero FJ Santos Sanz S Lopez Encinar P de la Torre Pardo MP Gil Gonzalez JM Factors associated with the appropriate use of preoperatory hospital stays: historical cohort study BMC Health Serv Res 2007 7 187 10.1186/1472-6963-7-187 18021440 PMC2212640

[B7] Ronen B Pliskin J Pass S Wiley Effects of Fluctuations, Variability, and Uncertainty on the System Focused Operations Management for Health Services Organizations 2006 Jossey-Bass a Wiley Inprint, San Francisco, CA 219 35

[B8] 10 High Impact Changes for service improvement and delivery: a guide for NHS Readers NHS Modernisation Agency 2011 http://www.ogc.gov.uk/documents/Health_High_Impact_Changes.pdf

[B9] Black D Pearson M Average length of stay, delayed discharge and hospital congestion Br Med J 2002 325 610 1 10.1136/bmj.325.7365.610 12242160 PMC1124147

[B10] Zwarenstein M Goldman J Reeves S Interprofessional collaboration: effects of practice-based interventions on professional practice and healthcare outcomes Cochrane Database Syst Rev 2009 8 CD000072 19588316 10.1002/14651858.CD000072.pub2

[B11] Blegen MA Sehgal NL Alldredge BK Gearhart S Auerbach AA Wachter RA Improving safety culture on adult medical units through multidisciplinary teamwork and communication interventions: the TOPS Project Postgrad Med J 2010 86 729 33 10.1136/qshc.2008.031252rep 21106808

[B12] Proudlove N Boaden R Jorgensen J Developing bed managers: the why and the how J Nurs Manag 2007 15 34 42 10.1111/j.1365-2934.2006.00632.x 17207005

[B13] Salazar A Gestión centralizada de los ingresos hospitalarios: modelo de priorización de camas Emergencias 2008 2 suppl 1 1 3

[B14] Improvement Leader’s Guide Improving flow. NHS Modernisation Agency 2011 http://www.internetgroup.ca/clientnet_new/docs/NHS%209%20-%20Improving%20Flow.pdf

[B15] Mahaffey S Optimizing patient flow in the enterprise. Hospitals can combine process management with information technology to redesign patient flow for maximum efficiency and clinical outcomes Health Manag Technol 2004 25 34 6 15328959

[B16] SPSS version 12.0 for Windows Computer program 2004 SPSS Inc, Chicago

[B17] Hospitales Top 20 Metodología Hospitales Top 20 Metodología. Iasist 2011 http://www.iasist.com/files/Metodologia%20indicadores.pdf

[B18] McDonagh MS Smith DH Goddard M Measuring appropriate use of acute beds.A systematic review of methods and results Health Policy 2000 53 157 84 10.1016/S0168-8510(00)00092-0 10996065

[B19] Hammond CL Pinnington LL Phillips MF A qualitative examination of inappropriate hospital admissions and lengths of stay BMC Health Serv Res 2009 9 44 10.1186/1472-6963-9-44 19265547 PMC2655293

[B20] Waiting for elective admissions Review of national findings 2003 National Health Service, UK

[B21] Ortiga B Capdevila C Salazar A Viso MF Bartolomé C Corbella X Effectiveness of a Surgery Admission Unit for patients undergoing major elective surgery in a tertiary university hospital BMC Health Serv Res 2010 10 23 10.1186/1472-6963-10-23 20096114 PMC2823739

[B22] Green LV Nguyen V Strategies for cutting hospital beds: the impact on patient service Health Serv Res 2001 36 421 42 11409821 PMC1089232

